# Microneedle‐Mediated Cell Therapy

**DOI:** 10.1002/advs.202304124

**Published:** 2023-10-30

**Authors:** Ziqi Gao, Tao Sheng, Wentao Zhang, Huiheng Feng, Jicheng Yu, Zhen Gu, Yuqi Zhang

**Affiliations:** ^1^ Zhejiang Provincial Key Laboratory for Advanced Drug Delivery Systems College of Pharmaceutical Sciences Zhejiang University Hangzhou 310058 China; ^2^ Liangzhu Laboratory Zhejiang University Medical Center Hangzhou 311121 China; ^3^ Jinhua Institute of Zhejiang University Jinhua 321299 China; ^4^ Department of General Surgery Sir Run Run Shaw Hospital School of Medicine Zhejiang University Hangzhou 310016 China; ^5^ National Key Laboratory of Advanced Drug Delivery and Release Systems Zhejiang University Hangzhou 310058 China; ^6^ MOE Key Laboratory of Macromolecular Synthesis and Functionalization Department of Polymer Science and Engineering Zhejiang University Hangzhou 310027 China; ^7^ Department of Burns and Wound Care Center Second Affiliated Hospital School of Medicine Zhejiang University Hangzhou 310009 China

**Keywords:** cell therapy, drug delivery, immunotherapy, microneedles, tissue regeneration

## Abstract

Microneedles have emerged as a promising platform for transdermal drug delivery with prominent advantages, such as enhanced permeability, mitigated pain, and improved patient adherence. While microneedles have primarily been employed for delivering small molecules, nucleic acids, peptides, and proteins, recent researches have demonstrated their prospect in combination with cell therapy. Cell therapy involving administration or transplantation of living cells (e.g. T cells, stem cells, and pancreatic cells) has gained significant attention in preclinical and clinical applications for various disease treatments. However, the effectiveness of systemic cell delivery may be restricted in localized conditions like solid tumors and skin disorders due to limited penetration and accumulation into the lesions. In this perspective, an overview of recent advances in microneedle‐assisted cell delivery for immunotherapy, tissue regeneration, and hormone modulation, with respect to their mechanical property, cell loading capacity, as well as viability and bioactivity of the loaded cells is provided. Potential challenges and future perspectives with microneedle‐mediated cell therapy are also discussed.

## Graphical abstract

The development of microneedles provides a new mode of therapeutic cell delivery for cell therapy. This perspective highlights the applications of microneedle‐mediated cell therapy in immunotherapy, regeneration medicine, and hormone modulation and discusses their mechanical properties, cell loading capacity, and viability of cells. Potential challenges and future prospects in this field are also discussed.

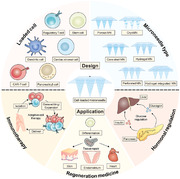

## 1 Introduction

Microneedle (MN)‐based transdermal delivery systems have attracted increasing interest as an alternative to conventional administration such as oral and parenteral routes over the past decades.^[^ ^1^, ^2^ ^]^ By physically penetrating the stratum corneum barrier to generate micropores, MNs facilitate the efficient transportation of therapeutic substances into the underlying dermal layers, ranging from small molecular drugs to nucleic acids, peptides, and proteins.^[^ ^3^, ^4^, ^5^ ^]^ Moreover, the micro‐sized needles could alleviate the discomfort during administration, and enhance patient safety compared to conventional hypodermic needles.^[^ ^6^ ^]^ Accounting for their prominent metrics including minimal invasiveness, favorable therapeutic efficacy, and improved patient adherence, MNs have been widely explored in various applications including drug delivery, vaccination, cosmetic products, and first aid interventions.^[^ ^1^, ^7^, ^8^, ^9^, ^10^, ^11^, ^12^ ^]^

In addition to facilitating transdermal transport of traditional molecular drugs, MN devices have also been investigated to deliver therapeutic cells for cellular therapy during the past decade.^[^ ^13^ ^]^ Cell therapy aims to leverage exogenous living cells such as immune cells, stem cells, and pancreatic cells to directly interact, express cytokines, or eliminate dysfunctional/diseased cells for modulating the function of the patient's cells. It is an evolving field that offers prospective treatment options for a wide range of diseases, including cardiovascular diseases, neurological disorders, and autoimmune disorders as well as certain types of cancer.^[^ ^14^, ^15^, ^16^, ^17^ ^]^ However, the therapeutic benefits of cell therapy critically hinge on the efficacious delivery of therapeutic cells to the designated target sites.^[^ ^18^, ^19^, ^20^ ^]^ Usually, systematically administered cells lack tissue targeting ability, thus resulting in suboptimal therapeutic efficacy and potential side effects. In addition, the instability and immune rejection of exogenous cells restrict the survival and functionality of administered cells in the body.^[^ ^21^, ^22^, ^23^, ^24^ ^]^ These challenges hinder the clinical translation of relevant cell therapy, especially for localized diseases such as solid tumors, skin disorders, and organ‐specific diseases.^[^ ^25^, ^26^, ^27^, ^28^ ^]^

**Figure 1:**
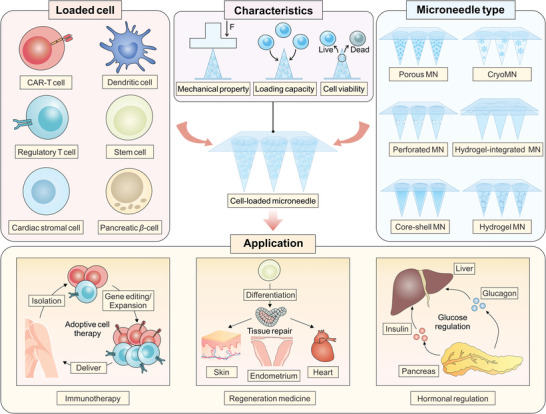
Microneedle‐facilitated cell delivery for immunotherapy, regeneration medicine, and hormonal regulation.

In this perspective, we introduce the recent advances in MN‐mediated cell therapy, with particular focus on its applications in immunotherapy, tissue regeneration, and hormonal regulation (**Figure** **1**). The assistance of MN in cell delivery could augment therapeutic effects through facilitating cell penetration and retention, enhancing tissue targeting, and reducing systematic toxicity.^[^ ^25^, ^26^ ^]^ The properties of representative MN systems are summarized in terms of fabrication methods, mechanical properties, cell loading capacity, as well as viability and bioactivity of the loaded cells. Finally, the key challenges and perspectives on future development of MN‐mediated cell therapy are also discussed.

**Table 1:** Summary of representative microneedles for cell therapy regarding their designs, properties including mechanical strength and loading capacity, and strategies for the maintenance of cell viability. NA: not applicable.

Diseases	Loaded cells	Type of MNs	Materials of MNs	Fabrication method	Mechanical strength	Loading capacity	Means of maintaining cell viability	Reference
Solid tumors	CAR T cells	Porous MN	PLGA	Molding with etching to form porous structure	2.4 N	3.4×10^6^ cells/cm^2^	Protect cells in the pores of the MN	[25]
Depending on the types of cells	HeLa cells expressing red fluorescent protein, keratinocytes, normal dermal fibroblasts, MSCs, melanocytes, T cells, and DCs	Cryomicroneedle	Frozen PBS with DMSO and sucrose	Stepwise cryogenic micromolding of cryogenic medium with pre‐suspended cells	0.17 N	Ranging from ∼8.9×10^5^ cells/cm^2^ to ∼4.3×10^7^ cells/cm^2^	Optimize cryogenic medium	[41]
Psoriasis	Treg cells	Perforated MN	Poly(VP‐*co*‐MMA), T cell culture medium with gelatin solution	“Sandwich” molding with top and bottom PDMS molds (molded from 3D‐printed male molds) to form perforated structure	0.376 N	3.6×10^5^ cells/cm^2^	1) Use thermo‐reversible gelatin as the cell loading matrix 2) Utilize hydrophobic MN shell to avoid nutrients depletion and unfavorable environment 3) Design channels on the MN shell for cell migration	[26]
Skin wounds	MSCs	Core‐shell MN	PLGA, GelMA	Two‐step molding of PLGA solution and GelMA‐MSC mixture (GMM)	> 1 N	∼2×10^3^ cells/cm^2^	1) Use GelMA for nutrients and oxygen exchange 2) Add cell media for preparing pre‐polymer solution in the process of making the GMM to ensure the nutrient supply 3) Vary the GMM curing time	[62]
Diabetic ulcer	ADSCs	Hydrogel MN	HAMA	Molding of HAMA, HMPP, PDGF‐D, re‐suspending ADSCs and gelatin as tips and pure gelatin as basal side	∼2 N	NA	1) Optimize the HAMA curing time 2) Add bioactive agent PDGF‐D to preserve cell viability	[63]
Endometrial injury	UCA‐PSCs	Hydrogel MN	GelMA	Molding of stem cells in GelMA as tips and nanozymes in GelMA as basal side	0.3 N	NA	Use GelMA for nutrients and oxygen exchange	[65]
Asherman's syndrome	En‐ADVs	Hydrogel MN	GelMA	Molding and seeding an En‐ADV suspension to form 3D cell spheroids on the MN patch	> 0.2 N	NA	1) Use GelMA for nutrients and oxygen exchange 2) Design microwells on the MN for better En‐ADVs culture as 3D spheroids	[66]
Myocardial infarction	Cardiac stromal cells	Hydrogel‐integrated MN	PVA, fibrin gel	Molding of PVA solution as tips and CSCs in fibrin gel as basal side	∼2 N	4×10^6^ cells/cm^2^	1) Use fibrin gel as cell reservoir 2) Get nutrients from the heart through MN channel	[67]
Myocardial infarction	iPSCs	Hydrogel MN	CNT, GelMA	Molding by layer‐by‐layer deposition onto the template	∼0.105 MPa	NA	Use GelMA as pregel solution for nutrients and oxygen exchange	[68]
Type 1 Diabetes	Pancreatic *β*‐cells	Hydrogel‐integrated MN	GSA, *α*‐amylose, crosslinked HA matrix, and alginate microgels with RGD and type IV collagen	Molding of the MN patch and encapsulation of *β*‐cells	0.18 N	Packing density: 2×10^6^ cells/mL in the alginate microgels	Encapsulate cells in the alginate microgels with RGD and type IV collagen to provide a matrix with biomimetic cell‐extracellular matrix adhesive interactions	[81]
Vitiligo	Melanocyte, keratinocyte, and mixed epidermal cell suspensions	Hollow MN	Silicon	Photolithography and deep silicon etching	NA	NA	Increase the dimensions of the microchannels to reduce the sheer forces	[83]
Depending on the types of cells	HaCaT cells, Dermal papilla cells	Hydrogel MN	Poly‐methyl methacrylate	Two‐stage micromolding and cell transplantation process	∼0.44 N	NA	Use collagen hydrogel to mimic the microenvironment of the extracellular matrix	[84]

## 2 Cell‐loaded microneedles for immunotherapy

**Figure 2:**
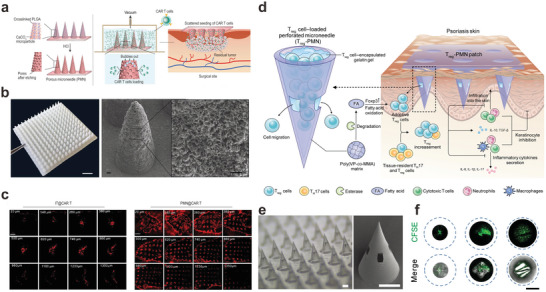
Cell‐loaded microneedles for immunotherapy. a) Scheme illustrating the fabrication of porous polymeric MN, CAR T cell loading, and implantation within the tumor bed after surgery. b) Photograph (left) and scanning electron microscopy (SEM) image (right) of the porous PLGA MN. Scale bars, 2 mm, 50 µm (left to right). c) Distribution of CAR T cells (red) within the tumor after single needle intratumoral injection (left) and porous MN insertion (right) into different layer depths. Scale bars, 1 mm. CAR T cells were pre‐labeled with DiI. Adapted with permission.^[^ ^25^ ^]^ Copyright 2022, Oxford University Press. d) Schematic illustration of the perforated MN‐mediated adoptive Treg therapy for psoriasis treatment. e) Photograph (left) and SEM image (right) of the perforated MNs. Scale bars, 300 µm. f) Confocal images of carboxyfluorescein diacetate succinimidyl ester‐labeled Treg cells encapsulated in perforated MN. Scale bar, 200 µm. Adapted with permission.^[^ ^26^ ^]^ Copyright 2023, AAAS.

Adoptive cell therapy, also known as cellular immunotherapy, has demonstrated prospective clinical advantages in patients who have failed conventional treatments against cancer and autoimmune diseases. It is a personalized approach that involves the isolation of immunocompetent cells, in vitro expansion and functional identification, followed by reinfusion to patients to directly eliminate the targeted cells or stimulate the defensive immune response.^[^ ^29^, ^30^ ^]^ For instance, chimeric antigen receptor (CAR) T cells can directly target and attack tumor cells by genetically engineering autologous T cells with synthetic receptors to recognize the specific surface antigens, which has yielded remarkable success in clinic for the treatment of hematological malignancies including leukemia and lymphoma.^[^ ^31^, ^32^, ^33^ ^]^ Unfortunately, their applications in solid tumors encounter additional challenges due to the complex tumor microenvironment and the presence of physical barriers that limit the efficacy of CAR T cells. Specifically, the abnormal vasculature, dense extracellular matrix, and interstitial fluid pressure impede the penetration of CAR T cells into the tumor bed, thus diminishing the antigen recognition and therapeutic effects.^[^ ^25^, ^33^, ^34^, ^35^ ^]^ To improve the infiltration into the solid tumor, Li et al. proposed a porous polymeric MN patch for deep delivery of CAR T cells (**Figure** **2a** **‐c**).^[^ ^25^ ^]^ MNs with micro/nano‐scale porous structure could be formulated by either an etching method or direct assembly from nanoporous materials.^[^ ^36^ ^]^ In their work, the porous MN was fabricated by etching the calcium carbonate particles embedded in MN made of the poly(lactic‐*co*‐glycolic acid) (PLGA) matrix. The obtained MN maintained sufficient mechanical strength to penetrate into the tumor (2.4 N/needle), which could be ascribed to the cross‐linked structure and the high molecule weight of PLGA in the formulation. The porous structure allowed loading up to 22000 CAR T cells per needle, meanwhile the array of MNs enabled scattered distribution of cells throughout the solid tumor. In vivo studies demonstrated that the porous MN‐delivered CAR T cells exhibited enhanced infiltration and immune stimulation compared to direct intratumoral injection, effectively inhibiting tumor growth in both a post‐surgical resection melanoma model and an orthotopic pancreatic tumor model.

In addition to CAR T cell that can directly kill tumor cells, dendritic cell (DC) is a type of specialized antigen‐presenting cells serving as a vaccine to elicit immune responses in T cells against specific antigens, such as cancer cells or infectious agents.^[^ ^37^ ^]^ Previous studies indicated that the intradermal administration of the DC vaccine could induce stronger immune responses compared to other administration routes.^[^ ^38^ ^]^ However, the administration of vaccinations through the intradermal route generally necessitates the utilization of the Mantoux technique and mandates the expertise of skilled individuals. Nevertheless, the efficacy of Mantoux injections remains modest, with reported failure rates reaching as high as 70%, which is predominantly attributed to the challenge of precisely administering the vaccine into the intradermal layer.^[^ ^39^, ^40^ ^]^ Alternatively, Xu and coworkers designed a cryomicroneedle (cryoMN) patch for DC vaccination.^[^ ^41^, ^42^ ^]^ The patch was fabricated by solidification after a stepwise, gradient cryogenic process (from −20 °C to −196 °C) with optimized cryogenic medium (phosphate‐buffered saline (PBS) with 2.5% (vol/vol) dimethylsulfoxide (DMSO) and 100 mM sucrose), which could preserve the activity of the pre‐suspended cells.^[^ ^41^ ^]^ The prepared cryoMNs were preserved at low temperature before usage. When taken out from the cryopreservation environment (−196 °C liquid nitrogen), they began to melt after 140 s at room temperature (RT) and 60 s when placed on a fingertip. The ex vivo tests on the porcine skin demonstrated that the skin penetration ability decreased when the cryoMNs were exposed to RT for longer durations. At a residence time of 30 s at RT, the cryoMNs could reach the dermal layer (∼500  µm thick), and they could pierce through the stratum corneum of the porcine skin (∼20  µm thick) after exposure for 40 s. While the cryoMNs would lose the penetration ability if they stayed at RT for more than 50 s. As for the viability of the loaded DCs, over 70% of the cells remained after melting these fresh cryoMNs in PBS (37 °C). Upon insertion into the skin, the preloaded cells were released from the cryoMNs along with the melting of needles, and subsequently migrated and proliferated within the skin. In a mouse melanoma model, the cryoMN‐delivered ovalbumin pulsed DCs induced stronger antigen‐specific immune responses and significantly enhanced anti‐tumor capability compared to both intravenous and subcutaneous injections methods.

Regulatory T (Treg) cell is another subtype of T cells with immune suppressive functions to restrain the over‐activation of effector cells for maintaining immune homeostasis. The malfunctions of Treg cells have been implicated in numerous autoimmune diseases, including type I diabetes, psoriasis, and inflammatory bowel disease.^[^ ^43^, ^44^ ^]^ Adoptive transfer of Treg cells was reported to be capable of restoring immune surveillance and homeostasis in the disease lesion.^[^ ^45^, ^46^ ^]^ However, insufficient tissue targeting and local accumulation of Treg cells hinder the clinical translation of systemic Treg therapy, especially for the treatment of regional diseases. Gu, Zhang, and coworkers developed perforated MNs to locally deliver Treg cells with augmented immune suppression to treat autoimmune disease in a representative psoriasis model (Figure 2d‐f).^[^ ^26^ ^]^ The perforated MN was first formed by a “sandwich” molding method via squeezing the polymeric shell made of poly(vinyl propionate‐*co*‐methyl methacrylate) [poly(VP‐*co*‐MMA)] between the top and bottom polydimethylsiloxane (PDMS) molds. The ratio of VP and MMA was optimized to ensure the stiffness of the needle (0.376 N/needle). Next, the Treg cells in gelatin gel were further filled into the MN shell under vacuum to form the inner core. The introduction of gelatin as the cell loading matrix could maintain the survival of Treg cells for at least 6 h. The core‐shell structure of the perforated MN favored a spacious cell loading cavity and desired mechanical properties. Meanwhile, the featured channels on the MN shell with sizes of ∼146 µm in length and ∼82 µm in width allowed free cell migration into the skin tissue upon insertion, which could prevent nutrient depletion and potential cell death caused by the slow degradation of the MN matrix. Furthermore, the polymeric MN shell made of poly(VP‐*co*‐MMA) could generate fatty acids in the inflammatory lesions to reinforce the suppressive function of Treg cells via fatty acid oxidation‐mediated metabolic intervention. In contrast to the administration of cell injections via intravenous or intradermal routes, perforated MN‐mediated Treg cell therapy significantly alleviated the inflammatory symptoms of psoriasis in a psoriasis‐like mouse model.

## 3 Cell‐loaded microneedles for tissue regeneration

Stem cells, featured with self‐renewal capacity and ability to differentiate into various cell types, possess immense potential in the field of regenerative medicine for the treatment of osteoarthritis, stroke, cardiopathy, and cancer.^[^ ^47^, ^48^, ^49^, ^50^ ^]^ Moreover, the emergence of induced pluripotent stem cells (iPSCs), characterized by their ability to differentiate into different somatic cells and potential serving as an unlimited source of regenerative cells, has revolutionized stem cell research and potentialized cell therapies against intractable diseases. Therefore, the exploration of more rational and efficient methods for administering stem cells holds merit in the pursuit of achieving desirable therapeutic outcomes.^[^ ^51^, ^52^, ^53^, ^54^ ^]^ A common approach is through cell transplantation via direct injection or surgical implantation, often accompanied by tissue damage due to invasive trauma.^[^ ^55^ ^]^ In addition, cells delivered by these approaches often encounter obstacles regarding constrained residence time and unfavorable homing efficacy, predominantly attributed to the immune system or complex internal environment. In previous studies, MN systems were mainly employed in the delivery of small molecular drugs, immunomodulatory cytokines, and nanoparticles as well as exosomes to facilitate tissue regeneration.^[^ ^56^, ^57^, ^58^, ^59^, ^60^, ^61^ ^]^ Nevertheless, recent advancement of MN systems in cell delivery contributes to an evolution in the field of stem cell therapy. The MNs provide enhanced cell residence by penetrating the tissue and directly transporting cells to the targets with minimal damage. Furthermore, localized delivery could bypass systematic immune response and provide the feasibility of transplantation.

**Figure 3:**
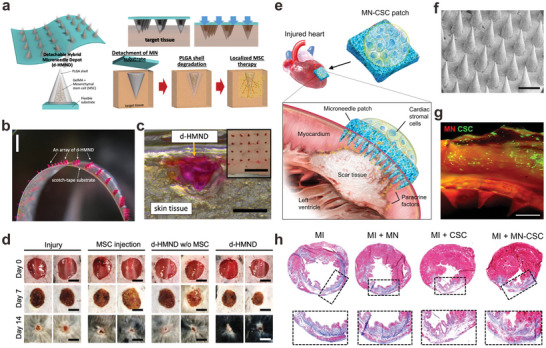
Cell‐loaded microneedles for tissue regeneration. a) Scheme of the detachable MN depot loading MCSs for wound healing. b) A representative image of the detachable MN depot with red‐dyed PLGA shell. Scale bar, 3 mm. c) Mouse skin after applying the detachable MN depot (rhodamine B as a visualizing agent). Scale bars, 500 µm and 3 mm (inset). d) Photographs of wound healing in different groups. Scale bars, 10 mm. Adapted with permission.^[^ ^62^ ^]^ Copyright 2020, Wiley‐VCH. e) Scheme of the overall design of the MN‐CSC patch for the treatment of infarcted heart. f) SEM image of the PVA MN array. Scale bar, 500 µm. g) Confocal image showing the distribution of DiO‐labeled CSCs (green) in the fibrin gel base of the cardiac patch. Scale bar, 500 µm. h) Masson's trichrome staining graphs showing the heart morphology and fibrosis three weeks after various treatments (red, viable tissue; blue, scar). Adapted with permission.^[^ ^67^ ^]^ Copyright 2018, AAAS.

In the field of wound repair, Lee et al. demonstrated a detachable hybrid MN depot (d‐HMND) for local delivery of mesenchymal stem cells (MSCs) to promote the healing process.^[^ ^62^ ^]^ In their system, the MN patch was composed of a solid PLGA shell with degradation time over two weeks, a gelatin methacryloyl (GelMA) core as the culture matrix for MSCs, and a detachable substrate made of scotch tape (**Figure** **3a** **‐c**). The GelMA matrix ensured nutrient supply for maintaining cell viability above 90% for 24 h. Once applied to the wound area, the detachable tape substrate was removed, leaving MN depots with concentrated MSCs at the injured sites to facilitate wound regeneration. In an excisional wound model, this hybrid cell patch exhibited elevated wound closure rates and improved re‐epithelialization, meanwhile minimizing the cell dosage required for the treatment (Figure 3d). In another work presented by Xu et al., a hydrogel‐based MN system was engineered for the delivery of adipose‐derived stem cells (ADSCs) to treat diabetic ulcer.^[^ ^63^ ^]^ For the fabrication of hydrogel MNs, micromolding and photolithographic process are generally used.^[^ ^64^ ^]^ The ADSCs loaded‐MNs were manufactured by UV polymerization of the methacrylated hyaluronic acid (HAMA) solution in the presence of the photoinitiator‐2‐hydroxy‐2‐methylpropiphenone (HMPP), which was premixed with ADSCs and platelet‐derived growth factor D (PDGF‐D). The obtained ADSCs‐loaded MNs exhibited desired mechanical strength (∼ 2 N/needle) and high cell viability (above 90% within 24 h). Moreover, the addition of bioactive PDGF‐D contributed to the augmented proliferation and enhanced function of ADSCs. This system could deliver ADSCs to relatively ductile wounds with minimal tissue damage. Upon insertion into the skin, hydrogel MNs could absorb large quantities of tissue fluid into the polymeric network, leading to MN swelling and cell release. In a full‐thickness skin excisional wound model of diabetic mice, ADSCs loaded‐MNs demonstrated expedited diabetic wound healing rates, condensed collagen deposition, as well as strengthened re‐epithelialization and angiogenesis.

Besides wound repair, Zhu et al. proposed a hybrid MN patch composed of needles loading umbilical artery‐derived perivascular stem cells (UCA‐PSCs) and antioxidant nanozymes CeO_2_ at the backing for in situ endometrial repair.^[^ ^65^ ^]^ The MNs formed by a 30% GelMA solution conferred sufficient mechanical strength to penetrate the endometrium (0.3 N/needle). Attributed to the biocompatibility and bioactivity of GelMA, high‐proportioned live cells could be observed after one month. With the assistance of the MN array, UCA‐PSCs could reach the injury sites effectively, promoting smooth muscle regeneration and neovascularization in the damaged endometrium. In addition, the embedded CeO_2_ at the backing layer further eliminated the excessive reactive oxygen species which might impede cell survival. Human endometrium‐derived adventitial cells (En‐ADVs), postulated to be innate progenitors of MSCs in the uterus, are also reported to be efficient in seeding cells for uterine regeneration. To deliver En‐ADV spheroids to the injured uterus, Li et al. put forward a hierarchical MN patch featuring microwells for culturing En‐ADVs as 3D spheroids.^[^ ^66^ ^]^ Compared with 2D monolayer cells, 3D dissociated En‐ADVs offered a microenvironment that mimicked in vivo conditions, therefore facilitating pluripotency maintenance, cell proliferation and migration, as well as angiogenesis. MNs prepared with 30% GelMA solution were fabricated to achieve sufficient mechanical strength (> 0.2 N/needle) and appropriate degradation time (degraded over 50% in one month). Considering that microorganism invasion often leads to the failure of embryo or stem transplantation, they also introduced lactoferrin (LF) in the GelMA MNs to counteract microbial invasion for more efficient intrauterine functional regeneration. In an Asherman's syndrome rat model, rats treated with the En‐ADV‐loaded MNs presented expedited uterine morphological regeneration and substantial restoration of the endometrial receptivity and reproductive function.

For the myocardial infarction (MI) treatment, Tang et al. proposed a hydrogel‐integrated MN system by incorporating cardiac stromal cells (CSCs) on the hydrogel base to release paracrine factors (Figure 3e‐h).^[^ ^67^ ^]^ The system was fabricated by a two‐step molding with aqueous polyvinyl alcohol (PVA) solution as the tips and the addition of CSCs in fibrin gel as the basal side. The PVA MNs functioned as the channels for communication between the cells and the host myocardium accounting for the ability to transport solute in the gel state, allowing CSCs to obtain nutrients from the host while releasing the paracrine factors to repair the myocardium. This microneedle patch integrated with cardiac stromal cells (MN‐CSCs) demonstrated beneficial effects in relieving myocardial apoptosis as well as promoting myocyte proliferation and angiogenesis in a rat MI model. Furthermore, heart morphology, fibrosis, and the left ventricular wall motion ability were also accelerated. In a porcine model of acute MI, the MN‐CSCs was capable to sustain the cardiac function without inducing toxicity. Apart from harnessing the paracrine effects of cells, which involves the communication of secretions between the transplanted cells and hosts through MNs, another strategy is to directly employ stem cells to replace the injured tissue. Sun et al. designed a multi‐layered cardiac patch with MN array loading vascular endothelial growth factor (VEGF) and interleukin‐10 (IL‐10) as the bottom layer, carbon nanotubes (CNTs) as the conductive middle layer, and iPSCs‐residing GelMA scaffold as the upper layer.^[^ ^68^ ^]^ The parallel‐aligned CNTs in the middle layer not only enhanced mechanical strength but also induced directional cell growth on the surface and provided a platform for electrical signaling between cells. Under suitable inducing conditions, iPSCs could be differentiated into cardiomyocytes (CMs) to restore autonomous beating capacity. The conductive patch allowed simultaneous contraction of CMs to keep synergies with the heart in vivo. When applied to a mouse MI model, this cardiac patch adhered to the injured site, then suppressed left ventricular wall thinning and displayed significant therapeutic effects in maintaining cardiac pump function.

## 4 Cell‐loaded microneedles for hormonal regulation

Hormones are chemical messengers produced by endocrine organs or tissues in the body, which play a vital role in regulating various physiological processes and maintaining homeostasis. However, aberrant hormone secretion may give rise to various diseases and disorders.^[^ ^69^, ^70^ ^]^ In the case of insulin, deficiency in its secretion could bring about diabetes and hyperglycemia, while excessive insulin can result in hypoglycemia.^[^ ^71^ ^]^ For patients with type 1 diabetes, pancreatic islet or whole pancreas transplantation is the most promising treatment currently available to restore *β*‐cell mass and maintain normoglycemia while simultaneously ameliorating hypoglycemia. Nevertheless, exogenous organ transplantation usually brings about severe immune rejection and requires the administration of immunosuppressant medications, which may accompany infectious diseases and digestive symptoms.^[^ ^72^, ^73^ ^]^

**Figure 4:**
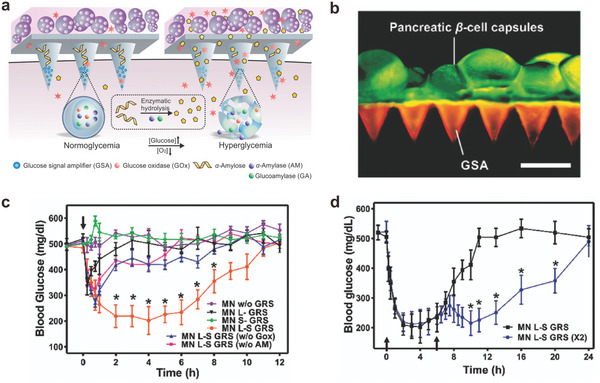
Cell‐loaded microneedles for hormonal regulation. a) Scheme of the glucose‐responsive system (GRS) based on an MN‐array patch integrated with pancreatic *β*‐cells and GSA. b) Fluorescence microscopy image of the live (cell‐based) and synthetic glucose‐responsive systems (L‐S GRS). Scale bar, 500 µm. c) Blood glucose levels of diabetic mice after treatment with empty MNs without GRS (w/o GRS), MNs integrated with only L‐GRS (L‐GRS), MNs integrated with only S‐GRS (S‐GRS), MNs integrated with L‐S‐GRS (L‐S GRS), MNs integrated with L‐S‐GRS but without GOx in S‐GRS (L‐S GRS (w/o GOx)), and MNs integrated with L‐S‐GRS but without *α*‐amylose in S‐GRS (L‐S GRS (w/o AM)) (*P < 0.05). d) Blood glucose levels of diabetic mice treated with additional MN (L‐S GRS) 6 h‐post administration (*P < 0.05). The black arrows indicate the administration points. Adapted with permission from Ref. [81].

Alternatively, an “artificial pancreas” that mimics the secretion function of pancreatic cells has been explored to regulate blood glucose levels, such as closed‐loop insulin pumps, glucose‐responsive delivery systems (e.g., “smart” insulin gel/MN patch), and stimuli‐activated synthetic *β* cell (e.g., electrical, light and glucose stimuli).^[^ ^71^, ^74^, ^75^, ^76^, ^77^, ^78^, ^79^, ^80^ ^]^ Gu and coworkers integrated islet *β*‐cells with an MN patch to enable insulin secretion directly on the skin rather than transplantation into the body (**Figure** **4a**).^[^ ^81^ ^]^ The islet *β*‐cells were encapsulated into the MN bases made of alginate microgels with Arg‐Gly‐Asp (RGD) and type IV collagen (Figure 4b), offering a biomimetic environment for the survival and growth of the cells. In the hyaluronic acid (HA) MN tips, glucose signal amplifier (GSA) nanoparticles were encapsulated to enhance the responsibility to blood glucose. To be more specific, GSA was a self‐assembled polymeric nanosized vesicle comprised of hypoxia‐sensitive materials containing glucose oxidase, *α*‐amylase, and glucoamylase. When the blood glucose level was elevated in vivo, the local hypoxia caused by the degradation of glucose via glucose oxidase promoted the dissociation of GSA, subsequently releasing *α*‐amylase and glucoamylase. The released *α*‐amylase hydrolyzed the *α*‐amylose in the MN into disaccharides and trisaccharides, which were further converted to glucose via glucoamylase. This amplified signal was transmitted to *β*‐cells through MNs and effectively triggered insulin secretion for modulating the blood glucose levels without causing potential risks of hypoglycemia (Figure 4c,d). This approach circumvented the challenging issues of pancreatic cell therapy associated with immune response and long‐term efficacy.

## 5 Challenges and future directions

In conclusion, the MN‐based tunable cell delivery platform offers exciting prospects such as spatially targeted delivery, evenly scattered distribution, augmented cell retention and activity, as well as reduced systematic toxicity for improving the therapeutic efficacy and broadening the application of cell therapy (**Table** **1**). These needles can be tailored in terms of dimensions, structures, materials, and formulations to achieve personalized drug release profiles. Besides, stimuli‐responsive materials can be engineered to fulfill on‐demand cell therapy. Also, additional therapeutic moieties, such as nutrients, immune checkpoint inhibitors, and cytokines, could be supplemented to extend the therapeutic window or impose synergistic effects. Despite the advancements and promising clinical implications across cosmetic, therapeutic, and diagnostic applications, additional efforts should be focused on the feasibility of MN for cell therapy in view of balancing the mechanical strength and cellular functionality, ensuring optimal loading capacity, as well as achieving scalable production feasibility and affordable cost for future clinical translation.^[^ ^82^ ^]^

First, it is crucial to optimize the mechanical properties of the MNs to ensure their structural integrity and mechanical strength while minimizing tissue damage. To be noted, the rigid nature of MNs for penetration and the desirable soft environment for cellular viability are intuitively contradicted, which may be overcome by unique MN design, such as the perforated structure. The delicate details of 3D printing technique have sparked new ideas for MN design, allowing for the creation of tailored internal and external structures.^[^ ^41^, ^70^, ^71^ ^]^ Second, delivering a sufficient amount of cells is essential to assure therapeutic effectiveness. Increasing the surface area of MNs to expand their loading capacity by changing the surface/volume area ratio may be an alternative option. Meanwhile, it is imperative to minimize cell wastage and mitigate potential contamination risks during the loading process to ensure precision and quality in manufacturing. Third, preserving or even augmenting the viability and activity of cells during the fabrication process as well as after insertion are critical concerns that require further investigation. To ensure the survival and optimal functioning of cells, a spacious cavity and temporary protection from the external surroundings could furnish cells with a relatively satisfactory environment for their growth. Furthermore, careful consideration is essential when selecting materials and fabrication methods for MNs in order to closely mimic the natural extracellular environment and preserve their intended functions. Protective reagents and/or supplementary nutrients can be involved to extend cell viability. Several factors such as temperature, oxygen content, presence of bacteria, and other environmental conditions should also be thoroughly taken into account. Additionally, long‐term stability, sterility, and scalability of cell‐loaded MNs should be considered for further Good Manufacture Practice.

In summary, the prospects for cell delivery through MN systems represent a significant potential in the field of cell therapy. This MN‐based platform technology also possesses therapeutic potential for personalized treatment with customized cell type, cell quantity, and administration route to meet the specific clinical requirements for individual patients. With the development of immunology, cellular biology, tissue engineering, and materials science, we will have an in‐depth understanding of the mechanisms of cell‐to‐cell interactions, which could further guide the design and translation of MN‐mediated cell therapy.

## Conflict of Interest

Z.G. is the co‐founder of Zenomics Inc. and ZCapsule Inc. Z.G. and Y.Z. are the co‐founders of *µ*Zen Pharma Co., Ltd., and the other authors declare no conflict of interest.
